# Clinical impact of neutropenia and febrile neutropenia in metastatic colorectal cancer patients treated with FOLFOXIRI/bevacizumab: a pooled analysis of TRIBE and TRIBE2 studies by GONO

**DOI:** 10.1016/j.esmoop.2021.100293

**Published:** 2021-10-22

**Authors:** D. Rossini, A. Boccaccino, A. Sbrana, F. Daniel, B. Borelli, A. Raimondi, D. Santini, V. Conca, G. Tomasello, S. Caponnetto, F. Marmorino, A. Zaniboni, A. Buonadonna, G. Masi, S. Lonardi, F. Pietrantonio, A. Falcone, A. Antonuzzo, C. Cremolini

**Affiliations:** 1 Unit of Medical Oncology 2, Azienda Ospedaliero-Universitaria Pisana, Pisa, Italy; 2 Department of Translational Research and New Technology in Medicine and Surgery, University of Pisa, Pisa, Italy; 3 Service of Pneumo-Oncology, Unit of Pneumology, Azienda Ospedaliero-Universitaria Pisana, Pisa, Italy; 4 Department of Surgical, Medical and Molecular Pathology and Critical Care Medicine, University of Pisa, Pisa, Italy; 5 Oncology Unit 1, Department of Oncology IOV – IRCCS, Padua, Italy; 6 Department of Medical Oncology, Fondazione IRCCS Istituto Nazionale dei Tumori, Milan, Italy; 7 Department of Medical Oncology, University Campus Bio-Medico, Rome, Italy; 8 UOC Oncologia Medica, Fondazione IRCCS Ca’ Granda – Ospedale Maggiore Policlinico, Milan, Italy; 9 Policlinico Umberto I, Oncologia B, Department of Radiological, Oncological and Pathological Sciences, La Sapienza University, Rome, Italy; 10 Medical Oncology Unit, Poliambulanza Foundation, Brescia, Italy; 11 Department of Medical Oncology, Centro Riferimento Oncologico (CRO) IRCCS, Aviano, Italy; 12 Oncology Unit 3, Veneto Institute of Oncology IOV-IRCCS, Padua, Italy; 13 Unit of Medical Oncology 1, Azienda Ospedaliero-Universitaria Pisana, Pisa, Italy

**Keywords:** metastatic colorectal cancer, FOLFOXIRI, neutropenia, febrile neutropenia, G-CSF, longitudinal toxicity over time

## Abstract

**Background:**

TRIBE and TRIBE-2 studies demonstrated higher benefit from FOLFOXIRI (fluorouracil, leucovorin, oxaliplatin, and irinotecan)/bevacizumab compared with FOLFIRI (fluorouracil, leucovorin, and irinotecan) or FOLFOX/bevacizumab as an upfront option for metastatic colorectal cancer patients, with more toxicities. We focused on the incidence and longitudinal dynamics of neutropenia and febrile neutropenia (FN) in the two studies, to evaluate their clinical relevance, the magnitude of impact of FOLFOXIRI/bevacizumab, and the role of risk factors in predicting their occurrence.

**Methods:**

The overall incidence of grade 3-4 (G3-4) neutropenia and FN, the time to their onset, the use of granulocyte colony-stimulating factor, and the association with risk factors were evaluated in the overall population and according to treatment arm. FN episodes were assessed by Multinational Association for Supportive Care in Cancer (MASCC) score.

**Results:**

Among 1155 patients, 568 (49%) received FOLFOXIRI/bevacizumab. Overall, 410 (35%) experienced G3-4 neutropenia and 70 (6%) FN, 21 (2%) at high risk. FOLFOXIRI/bevacizumab was associated with higher incidence of neutropenia (51% versus 21%, *P* < 0.001), FN (8% versus 4%, *P* = 0.02), and high-risk FN [18 (3%) versus 3 (1%), *P* = 0.015]. No related deaths were observed. The first episode of G3-4 neutropenia and FN occurred mainly in the first 2 months in both arms. Longitudinal analysis showed different patterns of evolution over cycles between the arms (*P* < 0.001) G3-4 neutropenia being more frequent in the first cycles with FOLFOXIRI/bevacizumab. Older patients (*P* = 0.01) and females (*P* < 0.001) had a significantly higher risk of G3-4 neutropenia. No significant interaction effect between arm and analysed risk factors in terms of risk of G3-4 neutropenia or FN was observed. The incidence of FN among older females receiving FOLFOXIRI/bevacizumab was 12%. Neither G3-4 neutropenia nor FN impaired efficacy in terms of overall response rate, progression-free survival, and overall survival.

**Conclusions:**

FOLFOXIRI/bevacizumab has a higher risk of G3-4 neutropenia and FN than doublets/bevacizumab. FN occurred in <10% of patients, mostly as low-risk episodes. A closer monitoring during the first 2 months is recommended; prophylactic use of granulocyte colony-stimulating factor may be considered for older females.

## Highlights

• FOLFOXIRI/bevacizumab is associated with a higher incidence of G3-4 neutropenia and FN than doublets/bevacizumab.
• FN occurred in <10% of patients receiving FOLFOXIRI/bevacizumab and mostly as low-risk episodes.
• Older patients and females show a significantly higher risk of G3-4 neutropenia.
• Longitudinal analysis of neutropenia showed different patterns of evolution with doublets versus triplet.
• Neither G3-4 neutropenia nor FN impaired treatment efficacy.

## Introduction

The combination of the triplet FOLFOXIRI (fluorouracil, leucovorin, oxaliplatin, and irinotecan) with the anti-angiogenic bevacizumab is a valuable first-line option for selected metastatic colorectal cancer patients.

The phase III TRIBE trial firstly proved the efficacy of this regimen compared with the doublet FOLFIRI (fluorouracil, leucovorin, and irinotecan) plus bevacizumab as first-line therapy,^1^^,^^2^ while the phase III TRIBE2 trial subsequently demonstrated that the upfront exposure to FOLFOXIRI plus bevacizumab then followed by the reintroduction of the same agents after disease progression provided long-term benefit when compared with the sequential exposure to modified FOLFOX (mFOLFOX6) plus bevacizumab followed by FOLFIRI plus bevacizumab after disease progression.^3^

A recent individual patient data-based meta-analysis of five randomized trials of FOLFOXIRI plus bevacizumab versus doublets (FOLFOX or FOLFIRI) plus bevacizumab confirmed a statistically significant and clinically relevant survival advantage for the upfront intensified treatment that was obviously associated with a higher incidence of chemotherapy-related adverse events.^4^

In particular, higher percentages of grade 3 or 4 (G3 or G4) diarrhoea, neutropenia, and febrile neutropenia (FN) were reported among patients receiving FOLFOXIRI plus bevacizumab.^1^^,^^3^^,^^4^ Myelotoxicity may be especially relevant from a clinical point of view since its complications, including FN, might be related to severe or fatal complications,^5^ and might cause treatment discontinuation or dose reductions, thus compromising treatment adherence and potentially hampering its feasibility and efficacy.^6^ In this regard, the opportunity to use granulocyte colony-stimulating factors (G-CSFs) as primary prophylaxis when choosing FOLFOXIRI plus bevacizumab as an upfront regimen is a debated issue.

The aim of the present analysis was to focus on the frequency and the timing of occurrence of neutropenia and FN among patients enrolled in the TRIBE and TRIBE2 study, in order to identify characteristics of patients more likely to experience these adverse events, and to describe the use of G-CSF in these study populations.

## Materials and methods

TRIBE and TRIBE2 are two randomized, open-label, multicenter, phase III trials for unresectable, previously untreated metastatic colorectal cancer patients. Patients aged 18-70 years with Eastern Cooperative Oncology Group performance status (ECOG PS) ≤2, and patients aged 71-75 years with ECOG PS = 0 were eligible. In the TRIBE study, 508 patients were randomized 1 : 1 to receive FOLFIRI/bevacizumab or FOLFOXIRI/bevacizumab up to 12 cycles of induction chemotherapy, both followed by maintenance with 5-fluorouracil/bevacizumab until disease progression, unacceptable toxicities, or consent withdrawal. In the TRIBE2 study, 679 patients were randomly assigned to receive FOLFOX/bevacizumab (arm A) or FOLFOXIRI/bevacizumab (arm B) up to eight cycles of induction chemotherapy, both followed by maintenance with 5-fluorouracil/bevacizumab; after first disease progression, arm A received FOLFIRI/bevacizumab, whereas arm B received FOLFOXIRI/bevacizumab, both followed by the same maintenance, until second disease progression, unacceptable toxicities, or consent withdrawal.

The incidence of G3-4 neutropenia and of FN, the time to the onset of these events, and the use of G-CSF were evaluated in the modified safety population, including all patients who had received at least one dose of the study medications with available toxicity data, and according to the treatment arm. Patients who received G-CSF as primary prophylaxis were excluded. The administration of G-CSF was considered as primary prophylaxis when given from the first cycle and continued through subsequent cycles of chemotherapy without previous G3-4 neutropenia, and/or FN.

National Cancer Institute Common Terminology Criteria for Adverse Events (NCI CTCAE), version 3.0 and 4.0 were adopted in the TRIBE and TRIBE2 study, respectively. G3-4 neutropenia was defined as an absolute neutrophil count <1000/ml of blood; FN was defined as the development of fever with a temperature >38.3°C or a sustained temperature equal or higher than 38°C with an absolute neutrophil count equal or lower than 1000/ml. All episodes of FN were classified according to the Multinational Association for Supportive Care in Cancer (MASCC) Risk Score, that considered several clinical factors in order to predict outcome of patients experiencing FN, stratifying them in low risk (MASCC score ≥21) and high risk (MASCC score <21).^7^ Supplementary Table S1, available at https://doi.org/10.1016/j.esmoop.2021.100293, summarizes MASCC score variables and points. According to the study protocols, clinical examination and blood tests were carried out and collected within 48 h before each cycle.

The time to the onset of adverse events was determined according to the Kaplan–Meier method, and curves were compared using the log-rank test. Hazard ratios (HRs) and 95% confidence interval (CI) were estimated with a Cox proportional hazard model.

Neutropenia was also longitudinally assessed over the first eight cycles of induction chemotherapy using the toxicity over time (ToxT) approach. Details of the statistical approach of ToxT have been fully described by Thanarajasingam et al.^8^

The association of the following characteristics with the occurrence of G3-4 neutropenia or FN was evaluated: age, ECOG PS, gender, previous adjuvant chemotherapy, previous radiotherapy, and presence of bone metastases. Older patients were defined as people aged ≥65 years. Odds ratios (ORs) and relative CI were estimated with a logistic regression model. Significant results were confirmed in the multivariate logistic-regression analyses. Subgroup analyses of FOLFOXIRI/bevacizumab versus doublets/bevacizumab for the occurrence of ≥G3 adverse events according to the above reported characteristics were done by using interaction tests. Overall survival (OS) and progression-free survival (PFS) analyses were determined according to the Kaplan–Meier method and survival curves were compared using the log-rank test. All statistical analyses were carried out using MedCalc version 14.8.1 (MedCalc Software Ltd, Ostend, Belgium) and SAS version 9.4 (SAS Institute, Inc., Cary, NC).

Patients' data were recorded in electronic case report forms and were reviewed by medical monitors. Every patient provided written informed consent. TRIBE and TRIBE2 studies were conducted in accordance with the Declaration of Helsinki. The two studies are registered on Clinicaltrials.gov as NCT00719797 and NCT02339116, respectively.

## Results

A total of 1175 patients were included in the modified safety population, 586 (50%) in the FOLFOXIRI/bevacizumab group and 589 (50%) in the doublets/bevacizumab group (254 assigned to FOLFIRI/bevacizumab and 335 to FOLFOX/bevacizumab). In total, 20 out of 1175 patients received G-CSF as primary prophylaxis according to investigators’ indication and were excluded from this analysis. Among the 1155 patients included, 568 received FOLFOXIRI/bevacizumab and 587 a doublet plus bevacizumab [333 (57%) FOLFOX/bevacizumab and 254 (43%) FOLFIRI/bevacizumab] as first-line therapy. Patients’ characteristics are summarized in Supplementary Table S2, available at https://doi.org/10.1016/j.esmoop.2021.100293.

Overall, 410 (35%) patients experienced G3-4 neutropenia, with a higher incidence in the FOLFOXIRI/bevacizumab group compared with the doublets/bevacizumab group (51% versus 21%; OR 3.9, 95% CI 3.03-5.1, *P* < 0.001). Moreover, 70 patients (6%) had at least 1 episode of FN, and a total of 79 episodes of FN were observed. FN was observed more frequently in the FOLFOXIRI/bevacizumab group compared with the doublets/bevacizumab group (8% versus 4%, OR 1.81, 95% CI 1.1-2.98, *P* = 0.02) (Table 1).

**Table 1:** Incidence of G3-4 neutropenia, febrile neutropenia, and episodes of high-risk febrile neutropenia according to treatment arm

Adverse events	Overall, *N* = 1155	FOLFOXIRI/bev, *N* = 568	Doublets/bev, *N* = 587	OR (95% CI)	*P*
	Events, *N* (%)	Events, *N* (%)	Events, *N* (%)		
G3-4 neutropenia	410 (35)	288 (51)	122 (21)	3.92 (3.03-5.08)	***<0.001***
Febrile neutropenia	70 (6)	44 (8)	26 (4)	1.81 (1.10-2.98)	0.02
High-risk febrile neutropenia	21 (2)	18 (3)	3 (1)	5.31 (1.38-20.37)	0.02

Bold/italic are statistically significant *P* values.

Bev, bevacizumab; CI, confidence interval; G, grade; *N*, number; OR, odds ratio.

FN events were classified according to the MASCC score. A total of 21 (2%) patients experienced high-risk FN episodes: 18 (3%) and 3 (1%) in the FOLFOXIRI/bevacizumab arm and in the doublets/bevacizumab arm, respectively (OR 5.31, 95% CI 1.38-20.37, *P* = 0.015) (Table 1). Only one patient discontinued the treatment due to prolonged neutropenia. No FN-related death was observed.

**Figure 1:**
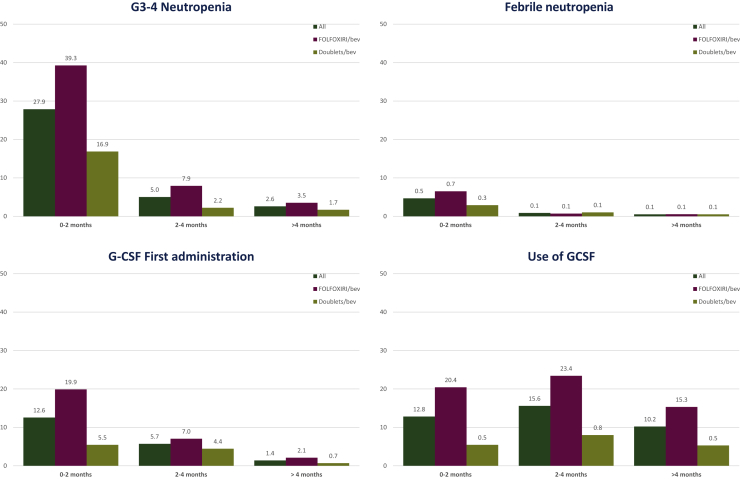
Histograms of G3-4 neutropenia, febrile neutropenia, G-CSF first administration, and overall use of G-CSF grouped for time periods. Bev, bevacizumab; FOLFOXIRI, fluorouracil, leucovorin, oxaliplatin, and irinotecan; G, grade; G-CSF, granulocyte-colony stimulating factor.

The time to the onset of G3-4 neutropenia was comparable between the two treatment arms, as the first episode occurred mainly in the first 2 months for both groups (median time: 1.0 versus 0.7 months for doublets/bevacizumab versus FOLFOXIRI/bevacizumab, HR 1.13, 95% CI 0.91-1.39, *P* = 0.27) (Supplementary Figure S1A, available at https://doi.org/10.1016/j.esmoop.2021.100293). Most G3-4 neutropenia episodes (78.5%) occurred during the first 2 months of treatment, whereas 14.2% of episodes occurred during the third or fourth month, and 7.3% after the fourth month (Figure 1).

**Figure 2:**
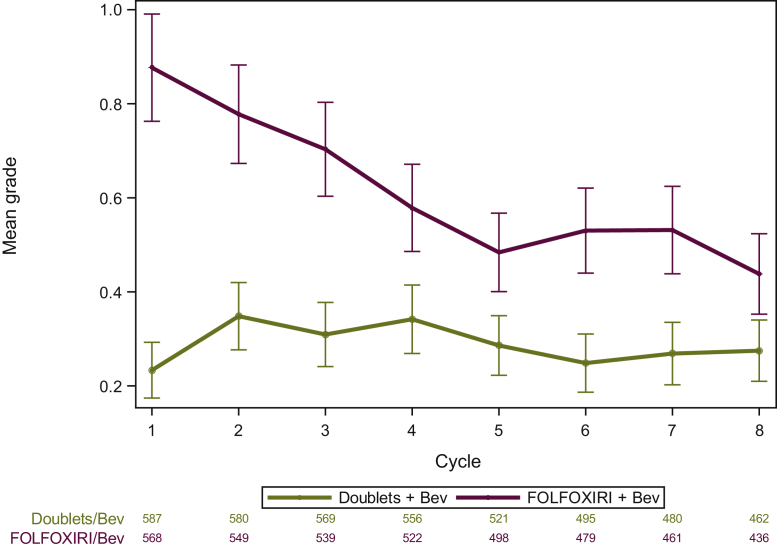
Longitudinal analysis of neutropenia grade over time by safety arm. FOLFOXIRI, fluorouracil, leucovorin, oxaliplatin, and irinotecan; Bev, bevacizumab.

Longitudinal assessment of any grade neutropenia is depicted in Figure 2 and Supplementary Figure S2, available at https://doi.org/10.1016/j.esmoop.2021.100293. The analysis of neutropenia grade over time showed different evolution over the cycles between the two arms (*P* < 0.001), and that the overall mean levels averaged over all time periods were higher for FOLFOXIRI/bevacizumab than doublets/bevacizumab (0.64 versus 0.30, *P* < 0.001) (Figure 2).

The time to occurrence of FN was numerically shorter among patients treated with FOLFOXIRI/bevacizumab than those receiving doublets/bevacizumab (0.6 versus 1.6 months, HR 1.46, 95% CI 0.89-2.40, *P* = 0.13) (Supplementary Figure S1B, available at https://doi.org/10.1016/j.esmoop.2021.100293). Most FN episodes (77.1%) occurred during the first 2 months; 14.3% occurred during the third or fourth month and 8.6% after the fourth month (Figure 1). Considering patients still on treatment after the fourth month [number (*N*) = 1018, 88.1%], only 2.9% of them experienced G3-4 neutropenia (*N* = 30) and <1% experienced FN.

PFS and OS were not significantly different among patients who experienced G3-4 neutropenia compared with the others [median PFS (mPFS): 11.7 versus 10.1 months; HR 0.91, 95% CI 0.80-1.03, *P* = 0.13; median OS (mOS): 27.9 versus 24.5 months; HR 0.90, 95% CI 0.78-1.04, *P* = 0.14] (Supplementary Figure S3A and B, available at https://doi.org/10.1016/j.esmoop.2021.100293), or among patients who experienced NF compared with the others (mPFS: 9.7 versus 10.8 months; HR 1.12, 95% CI 0.87-1.45, *P* = 0.38; mOS: 22.3 versus 26.1 months; HR 1.26, 95% CI 0.96-1.66, *P* = 0.09) (Supplementary Figure S3C and D, available at https://doi.org/10.1016/j.esmoop.2021.100293). No significant interaction effect for mPFS and mOS between treatment arm and G3-4 neutropenia (*P* = 0.83 and *P* = 0.99, respectively) or FN was observed (*P* = 0.26 and *P* = 0.41, respectively).

Patients who experienced G3-4 neutropenia had a higher response rate in the overall population (OR: 1.45, 95% CI 1.13-1.85, *P* = 0.003) and in the FOLFOXIRI/bevacizumab arm (OR: 1.60, 95% CI 1.14-2.26, *P* = 0.007), whereas there was no significant difference in the doublets/bevacizumab arm (*P* = 0.84). The *P* of interaction for the treatment arm was borderline significant (*P* = 0.06). The response rate was not different among patients developing FN or not in the overall population (*P* = 0.37), in the FOLFOXIRI/bevacizumab arm (*P* = 0.68), and in the doublets/bevacizumab arm (*P* = 0.59).

G-CSF was administered to 227 patients (20%); 165 in the FOLFOXIRI/bevacizumab arm (29%) and 62 in the doublets/bevacizumab arm (11%).

Consistently with the time to the onset of G3-4 neutropenia and FN, the first administration of G-CSF mainly occurred in the first 2 months (*N* = 145, 63.9%), whereas it was progressively less common during the subsequent months [during the third or fourth month: *N* = 66 (29.1%); after the fourth month: *N* = 16 (7%)] (Figure 1).

A total of 177 (15%) patients received G-CSF after their first episode of G3-4 neutropenia; 128 (23%) in the FOLFOXIRI/bevacizumab arm and 49 (8%) in the doublets/bevacizumab arm. Among them, 40 (3%) patients had a second episode of G3-4 neutropenia though receiving G-CSF as secondary prophylaxis, 32 (6%) and 8 (1%) in the FOLFOXIRI/bevacizumab and doublets/bevacizumab group, respectively (*P* = 0.30). Out of 27 (2%) patients who received G-CSF after their first episode of FN, 22 (4%) and 5 (1%) in the FOLFOXIRI/bevacizumab and doublets/bevacizumab group, respectively, only 2 (<1%) had a second episode of FN while on treatment with G-CSF (1 patient per arm, *P* = 0.34).

A total of 719 (62%) patients experienced a treatment delay in the induction phase: in 279 (39%) and 49 (7%) cases, delay was due to G3-4 neutropenia or FN, respectively. Treatment delays due to these toxicities were more frequent in the FOLFOXIRI/bevacizumab group (G3-4 neutropenia: 35% versus 13%, *P* < 0.001; FN: 6% versus 3%, *P* = 0.01).

Chemotherapy was administered at a reduced dose after the first episode of G3-4 neutropenia to 156 (14%) patients, 98 (17%) in the FOLFOXIRI/bevacizumab arm and 58 (10%) in the doublets/bevacizumab arm. Among them, 58 (5%) patients experienced a second episode of G3-4 neutropenia, 40 (7%) and 18 (3%) in the FOLFOXIRI/bevacizumab and doublets/bevacizumab arm, respectively (*P* = 0.29). Dose reduction after the first episode of FN was reported for 39 (3%) patients, 25 (4%) in the FOLFOXIRI/bevacizumab arm and 14 (2%) in the doublets/bevacizumab arm. Among them, 6 (1%) patients had a second episode of FN, 4 (1%) and 2 (<1%) in the FOLFOXIRI/bevacizumab arm and doublets/bevacizumab arm, respectively (*P* = 1.00).

Older age (*P* = 0.01), ECOG PS 0 (*P* = 0.01), and female gender (*P* < 0.001) were associated with a significantly higher risk of G3-4 neutropenia. These results were confirmed in the multivariate analyses. A trend towards higher risk was also found among patients previously exposed to adjuvant chemotherapy (*P* = 0.054) (Table 2).

**Table 2:** Incidence of G3-4 neutropenia and febrile neutropenia according to risk factors

Population *N* = 1155							
Risk factors	Number of patients	G3-4 neutropenia			Febrile neutropenia		
		Events, *N* (%)	OR (95% CI)	*P*	Events, *N* (%)	OR (95% CI)	*P*
Age							
≥65 years	408	164 (40.2)	1.37 (1.07-1.76)	***0.01***	28 (6.86)	1.24 (0.75-2.03)	0.40
<65 years	747	246 (32.9)	1		42 (5.62)	1	
ECOG PS							
1-2	144	38 (26.4)	0.62 (0.42-0.91)	***0.01***	10 (6.9)	1.18 (0.59-2.37)	0.64
0	1011	372 (36.8)	1		60 (5.9)	1	
Sex							
Female	481	214 (44.5)	1.95 (1.53-2.50)	***<0.001***	37 (7.7)	1.62 (1.00-2.63)	0.051
Male	674	196 (29.1)	1		33 (4.9)	1	
Bone metastasis							
Yes	39	9 (23.1)	0.54 (0.25-1.14)	0.10	5 (12)	2.38 (0.90-6.28)	0.08
No	1116	401 (35.9)	1		65 (5.8)	1	
Adjuvant chemotherapy							
Yes	79	36 (45.6)	1.57 (0.99-2.49)	0.054	3 (3.8)	0.59 (0.18-1.93)	0.39
No	1076	374 (34.8)	1		67 (6.2)	1	
Previous radiotherapy							
Yes	67	24 (35.8)	1.02 (0.61-1.70)	0.95	4 (6.0)	0.98 (0.35-2.78)	0.97
No	1088	386 (35.5)	1		66 (6.1)	1	

Bold/italic are statistically significant *P* values.

CI, confidence interval; ECOG PS, Eastern Cooperative Group Performance Status; G, grade; N, number; OR, odds ratio.

Females (*P* = 0.051) and patients with bone metastases (*P* = 0.08) were more likely to experience FN, though not significantly (Table 2).

**Figure 3:**
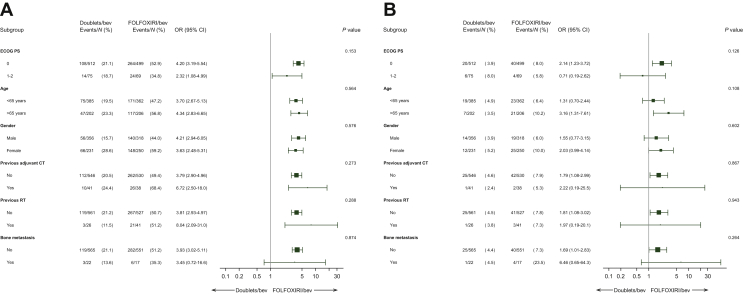
Forest plot of treatment effect on G3-4 neutropenia (A) and febrile neutropenia (B) according risk factors. Bev, bevacizumab; CI, confidence interval; CT, chemotherapy; ECOG PS, Eastern Cooperative Group performance status; FOLFOXIRI, fluorouracil, leucovorin, oxaliplatin, and irinotecan; G, grade; *N*, number; OR, odds ratio; RT, radiotherapy.

No significant interaction effect between treatment arm and analysed risk factors for G3-4 neutropenia or FN was observed (Figure 3A and B). The increased risk of developing FN with FOLFOXIRI/bevacizumab compared with doublets/bevacizumab, however, was more evident among females and older patients. Indeed, out of 162 older females, 79 (49%) experienced G3-4 neutropenia, 59 (63%) and 20 (29%) in the FOLFOXIRI/bevacizumab and in the doublets/bevacizumab arm, respectively (OR: 4.25, 95% CI 2.18-8.31, *P* < 0.001). Compared with all the other patients, older females had a higher risk of G3-4 neutropenia (OR: 1.90, 95% CI 1.36-2.66, *P* < 0.001).

A total of 13 (8%) older female patients had FN, 11 (12%) and 2 (3%) in the FOLFOXIRI/bevacizumab and doublets/bevacizumab arm, respectively (OR: 4.49, 95% CI 0.96-20.98, *P* = 0.06). No significant differences for FN were reported between older females and all the other patients (*P* = 0.26).

## Discussion

Neutropenia and FN are among the most frequent adverse events of cytotoxic agents. They may cause delay in treatment delivery or require dose modifications, eventually affecting dose-intensity.^5^^,^^9^, ^10^, ^11^, ^12^, ^13^ The risk of neutropenia and FN increases with combination regimens.^9^^,^^11^ Prevention and early management of neutropenia and FN are essential to avoid major complications (25%-30%), potentially life-threatening (up to 11%).^9^^,^^13^

The TRIBE and TRIBE2 studies demonstrated that FOLFOXIRI plus bevacizumab is a feasible and advantageous regimen as first-line treatment, and also as reintroduction after the evidence of disease progression, at the cost of a higher rate of adverse events including myelotoxicity.^1^^,^^3^

Our analysis confirms the higher incidence of G3-4 neutropenia and FN with FOLFOXIRI/bevacizumab compared with doublets/bevacizumab as first-line treatment of advanced colorectal cancer. The incidence of FN was relatively low (8%), however, also with the intensified chemotherapy. Moreover, FN episodes were mainly low-risk according to the MASCC score, and patients were mainly treated as outpatients with G-CSF and/or oral antibiotics. The onset of complications from FN was even more rare and no related deaths were reported, thus making the use of G-CSF as primary prophylaxis not recommended in the overall population according to current guidelines.^9^^,^^10^^,^^14^

The longitudinal evaluation of toxicity provides a more comprehensive description of adverse events, otherwise not identified by usual toxicity analyses. It confirms the overall higher incidence of neutropenia over time for FOLFOXIRI/bevacizumab and interestingly shows a different pattern of evolution according to treatment arm, the mean grade of neutropenia with FOLFOXIRI/bevacizumab being higher especially in the first cycles when compared with doublets/bevacizumab. The majority of G3-4 neutropenia and FN episodes were observed in the first 2 months of treatment independently of the treatment arm, as previously reported with other chemotherapy regimens, both for solid and haematological malignancies. This may be explained by the reactive management of the first episodes of myelotoxicity by clinicians, in terms of dose modifications and/or use of G-CSF as secondary prophylaxis.^12^^,^^15^^,^^16^ As shown by our data, following both dose reduction and/or G-CSF administration, the recurrence of G3-4 neutropenia and FN was very low.

Based on these results, a careful monitoring of neutropenia during the first months of treatment should be recommended to prevent FN and to properly manage subsequent treatment cycles. Consistent with literature data, older age and female gender were associated with a higher risk of G3-4 neutropenia, and a trend to a higher risk of FN was evidenced for females and for patients with bone metastases.^12^^,^^16^^,^^17^

A note of caution in the interpretation of data about the presumed higher risk of neutropenia for patients with better ECOG PS should be mentioned, due to the low number of patients with worse ECOG PS enrolled in the two studies. Furthermore, in many cases patients with worse ECOG PS received a lower number of cycles due to early progression or death, or in a few cases they started chemotherapy with reduced doses at the investigators’ choice. The increased risk of neutropenia and FN with FOLFOXIRI/bevacizumab was independent of the analysed risk factors, but appeared more relevant among older patients and females, thus suggesting a more careful monitoring, but also to consider a primary G-CSF prophylaxis in selected cases when the triplet is chosen as the upfront regimen.

Although G3-4 neutropenia and FN were associated with higher rates of treatment delay, this did not translate into lower overall response rate, or shorter PFS or OS. The administration of G-CSF as primary prophylaxis might be considered, however, when respecting dose intensity is more clinically relevant, such as in potentially resectable metastatic colorectal cancer patients, in the case of extensive disease in vital organs and/or symptomatic sites of metastasis, or in the neoadjuvant setting. Alternative schedules of FOLFOXIRI have also been investigated, especially in the Asian population, but no comparative efficacy data are available.^18^, ^19^, ^20^

*DPYD* (dihydropyrimidine dehydrogenase) and *UGT* (uridine 5′-diphospho-glucuronosyltransferase) variants were not tested before enrollment, as these pharmacogenomics analyses were not routinely carried out at that time. As shown by a previous analysis conducted on a large cohort of patients in the TRIBE study, those carrying relevant *DPYD*, and/or *UGT* polymorphisms experienced a higher rate of adverse events compared with the others, in particular G3-4 haematological adverse events, including neutropenia and FN.^21^ As a consequence, it can be hypothesized that genotype-guided dose modifications may decrease the rate of toxicities without affecting efficacy.^21^^,^^22^

### Conclusion

FOLFOXIRI/bevacizumab is associated with higher risk of G3-4 neutropenia and FN compared with doublets/bevacizumab. Most FN episodes were at low risk according to the MASCC score, and the overall incidence of FN was <10%, thus making the systematic use of G-CSF as primary prophylaxis not recommended in the overall population. Female gender and older age were risk factors for the development of these adverse events independently of the intensity of the upfront chemotherapy backbone, but especially relevant among patients receiving FOLFOXIRI/bevacizumab, thus leading to suggest the use of G-CSF as primary prophylaxis in this subgroup.
