# Supplementary material for: Clinical impact of neutropenia and febrile neutropenia in metastatic colorectal cancer patients treated with FOLFOXIRI/bevacizumab: a pooled analysis of TRIBE and TRIBE2 studies by GONO
Source: ESMO Open. 2021 Oct 22;6(6):100293. doi: 10.1016/j.esmoop.2021.100293 (PMC8551530; doi:10.1016/j.esmoop.2021.100293)
Supplement: Supplementary Tables [file mmc1.docx]

**Supplementary table 1: MASCC risk index factors and weights**

| **Characteristic** | **Weight** |
| --- | --- |
| Burden of febrile neutropenia with no or mild symptoms | 5 |
| No hypotension (systolic BP > 90 mmHg) | 5 |
| No chronic obstructive pulmonary disease | 4 |
| Solid tumor or hematological malignancy with no previous fungal infection | 4 |
| No rehydratation requiring parental fluids | 3 |
| Burden of febrile neutropenia with moderate symptoms | 3 |
| Outpatient status | 3 |
| Age <60 years | 2 |

Burden of febrile neutropenia refers to general clinical status as influenced by the febrile neutropenic episode. It is evaluated according to the following scale: No symptoms or mild symptoms (5), moderate symptoms (3), severe symptoms or moribund (0). Chronic obstructive pulmonary disease refers to active chronic bronchitis, emphysema, decrease in FEVs, need for oxygen therapy and/or steroids and/or bronchodilators. Previous fungal infection means demonstrated or suspected and empirically treated fungal infection. The points attributed to "burden of febrile neutropenia" are not cumulative.

| **Characteristics of patients**  **(Population *N*=1155)** | | | |
| --- | --- | --- | --- |
|  | **Number of patients** | **FOLFOXIRI/bevacizumab**  *N* (%) | **Doublets/bevacizumab**  *N* (%) |
| **Age** | | | |
| ≥65 years | 408 | 202 (49.5) | 206 (50.5) |
| <65 years | 747 | 362 (48.5) | 385 (51.5) |
| **ECOG PS** | | | |
| 1-2 | 144 | 69 (47.9) | 75 (52.1) |
| 0 | 1011 | 499 (49.4) | 512 (50.6) |
| **Sex** | | | |
| Female | 481 | 250 (52) | 231 (48) |
| Male | 674 | 318 (47.2) | 356 (52.8) |
| **Bone metastasis** | | | |
| Yes | 39 | 17 (43.6) | 22 (56.4) |
| No | 1116 | 551 (49.4) | 565 (50.6) |
| **Adjuvant chemotherapy** | | | |
| Yes | 79 | 38 (48.1) | 41 (51.9) |
| No | 1076 | 530 (49.3) | 546 (50.7) |
| **Previous radiotherapy** | | | |
| Yes | 67 | 41 (61.2) | 26 (38.8) |
| No | 1088 | 527 (48.4) | 561 (51.6) |

**Supplementary table 2: characteristics of patients.**

*N*, number; ECOG PS, Eastern Cooperative Group Performance Status.
