# Supplementary figures and images for: Clinical impact of neutropenia and febrile neutropenia in metastatic colorectal cancer patients treated with FOLFOXIRI/bevacizumab: a pooled analysis of TRIBE and TRIBE2 studies by GONO
Source: ESMO Open. 2021 Oct 22;6(6):100293. doi: 10.1016/j.esmoop.2021.100293 (PMC8551530; doi:10.1016/j.esmoop.2021.100293)

A

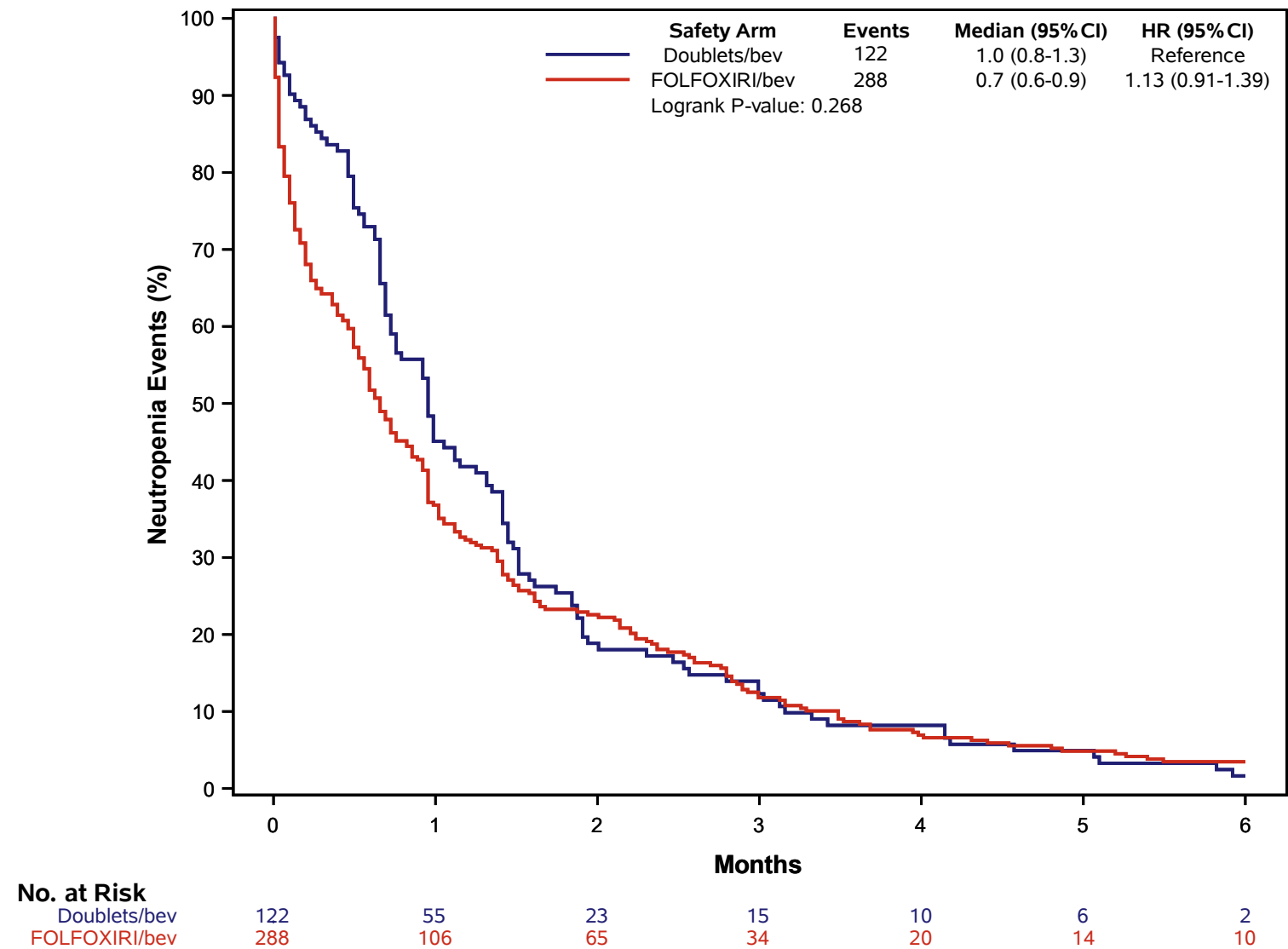

B

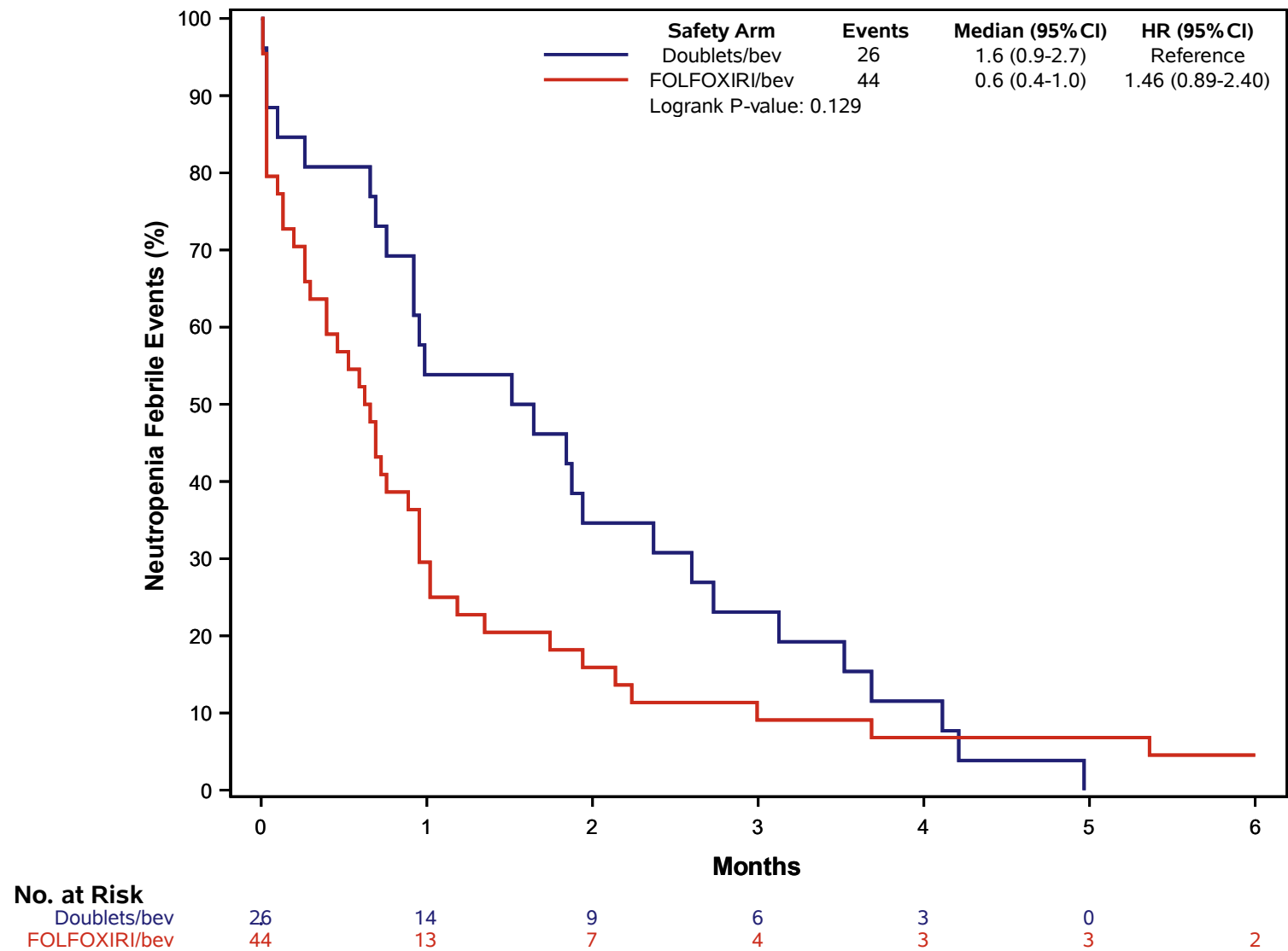

Supplement: Supplementary Figure S1 [file mmc2.pdf]

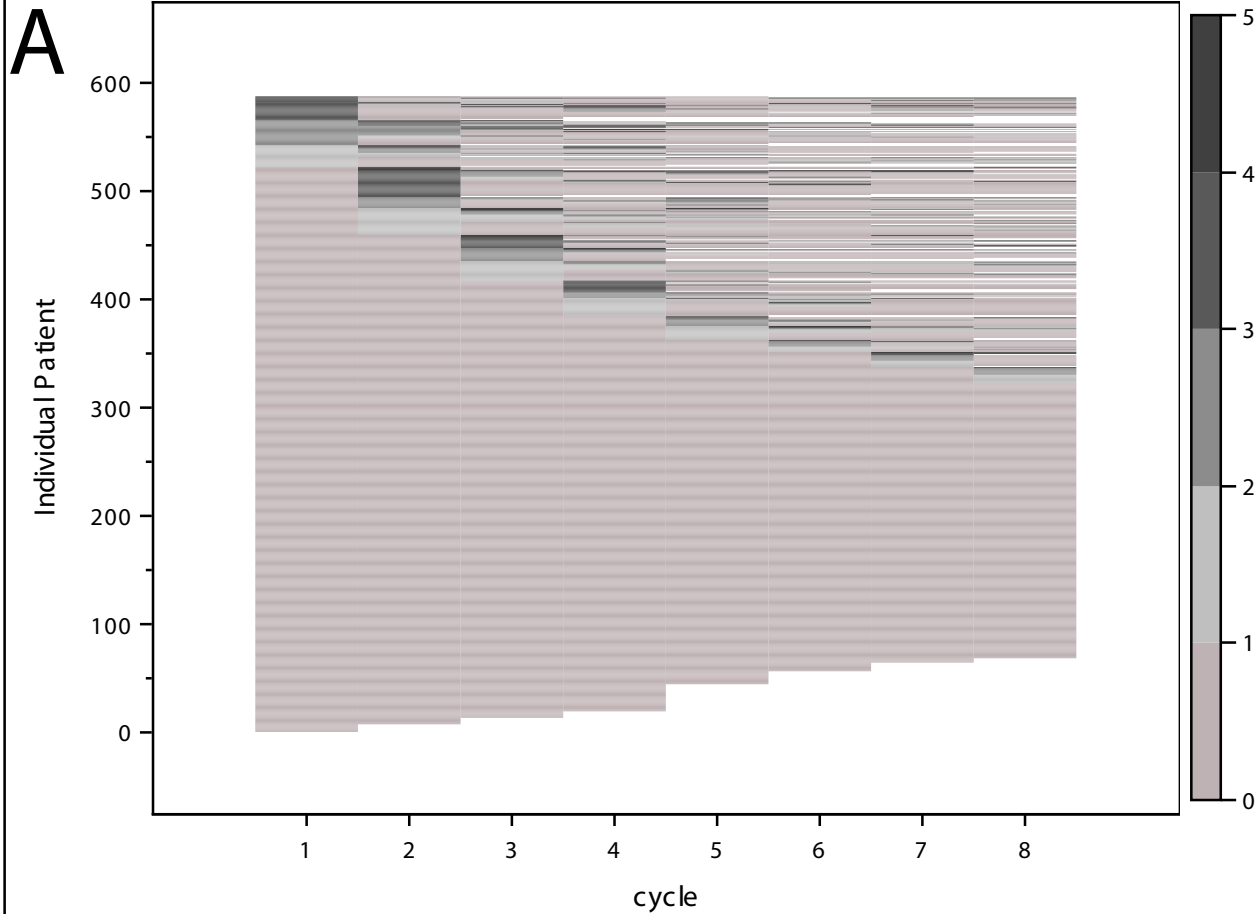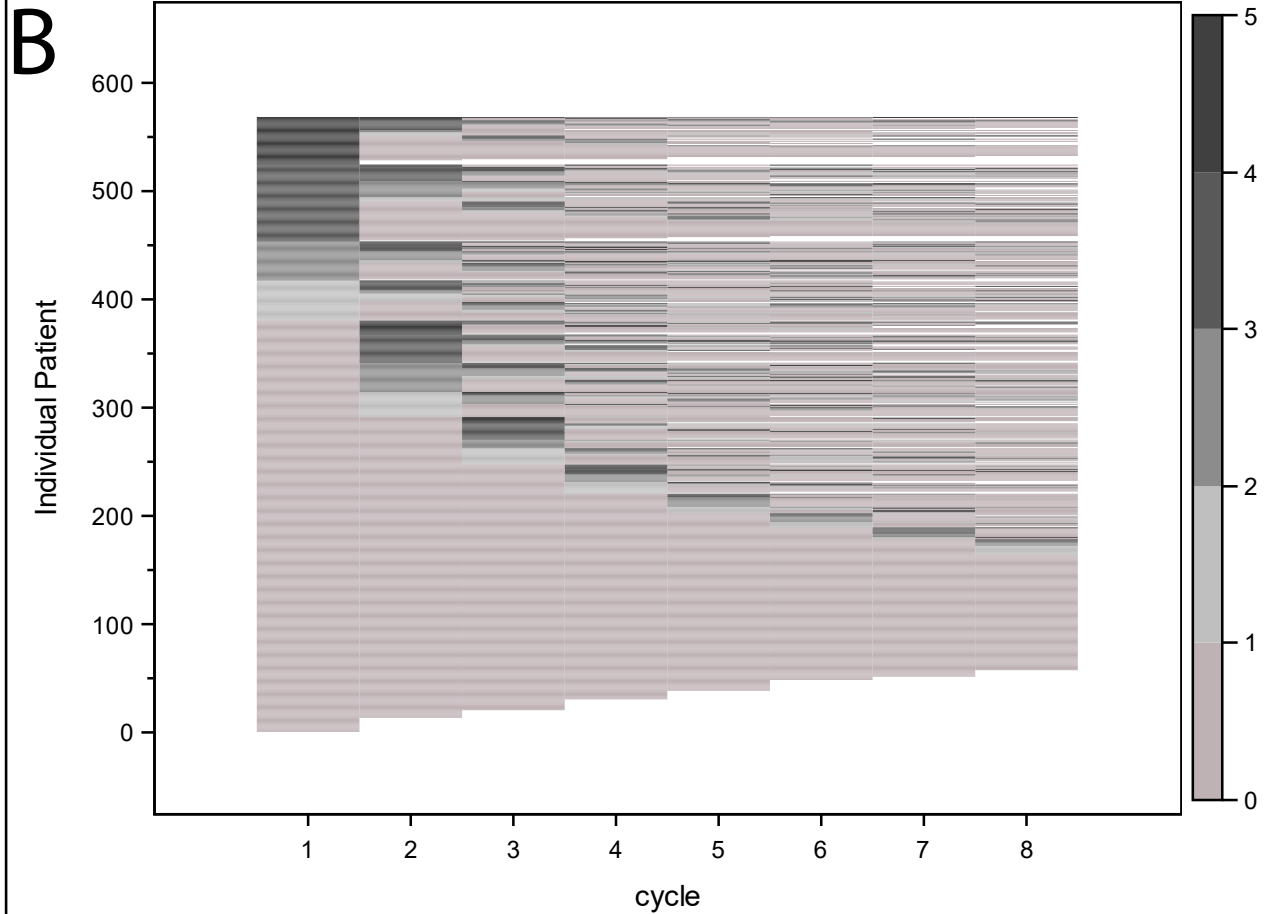

Supplement: Supplementary Figure S2 [file mmc3.pdf]

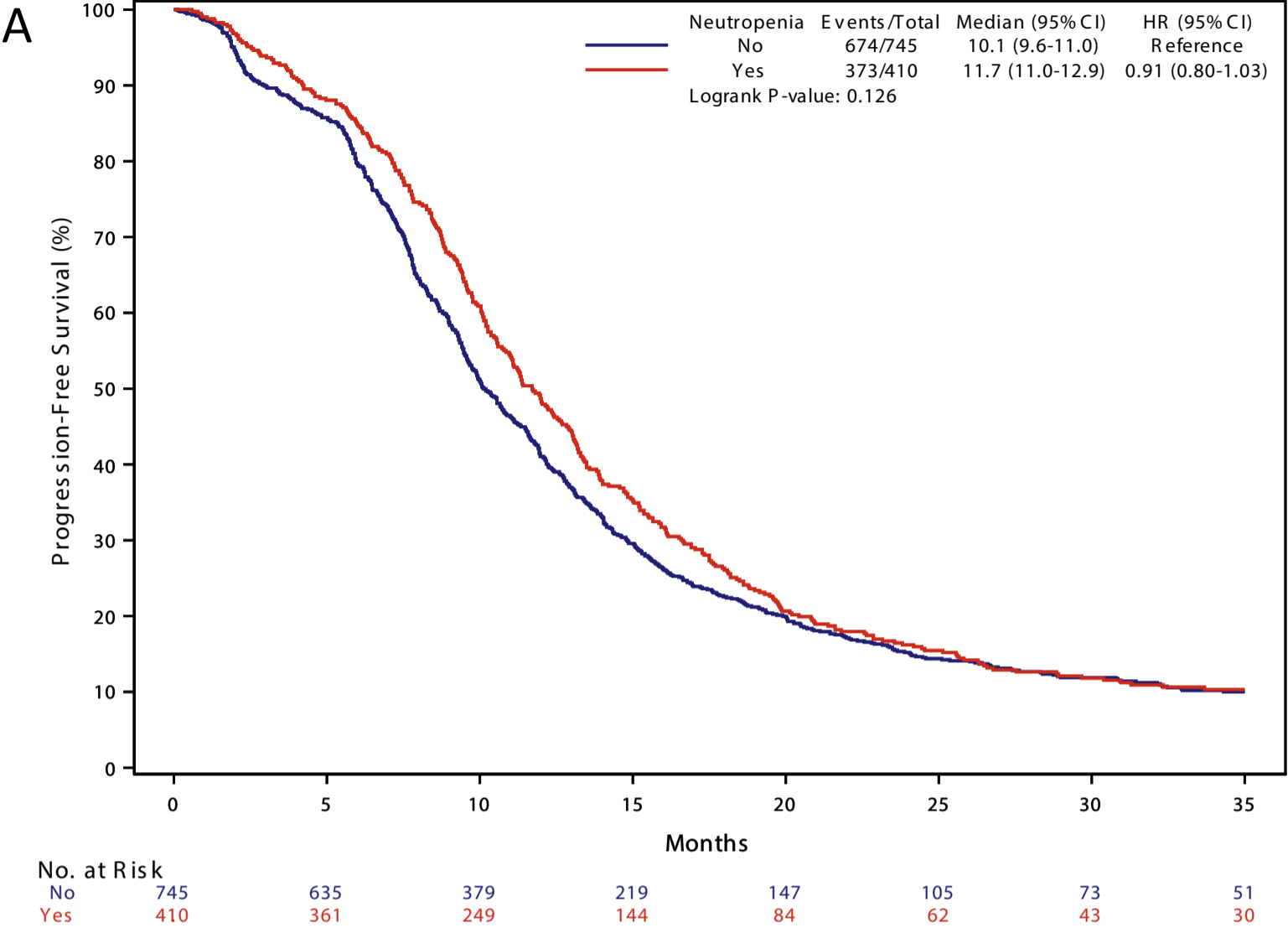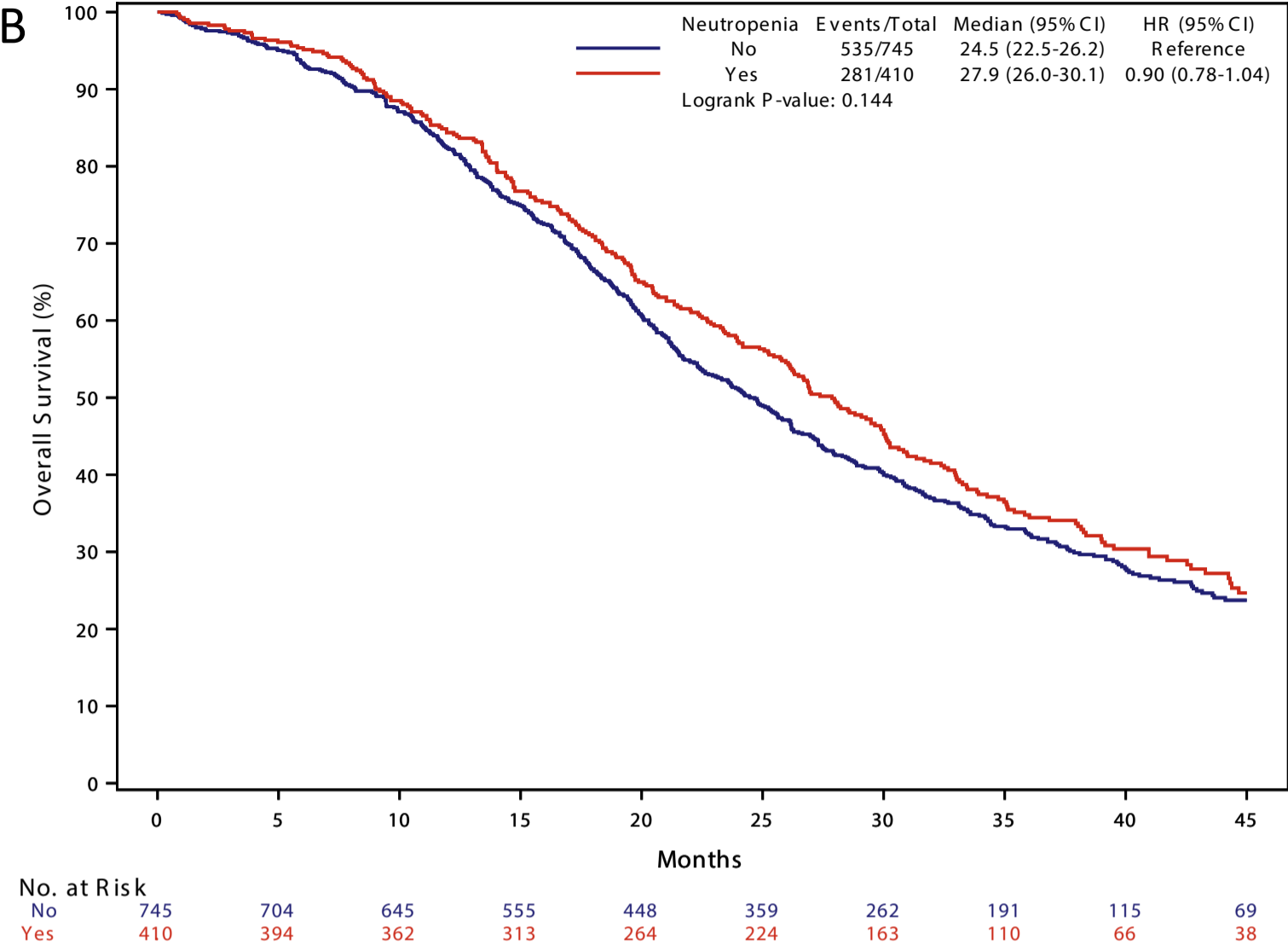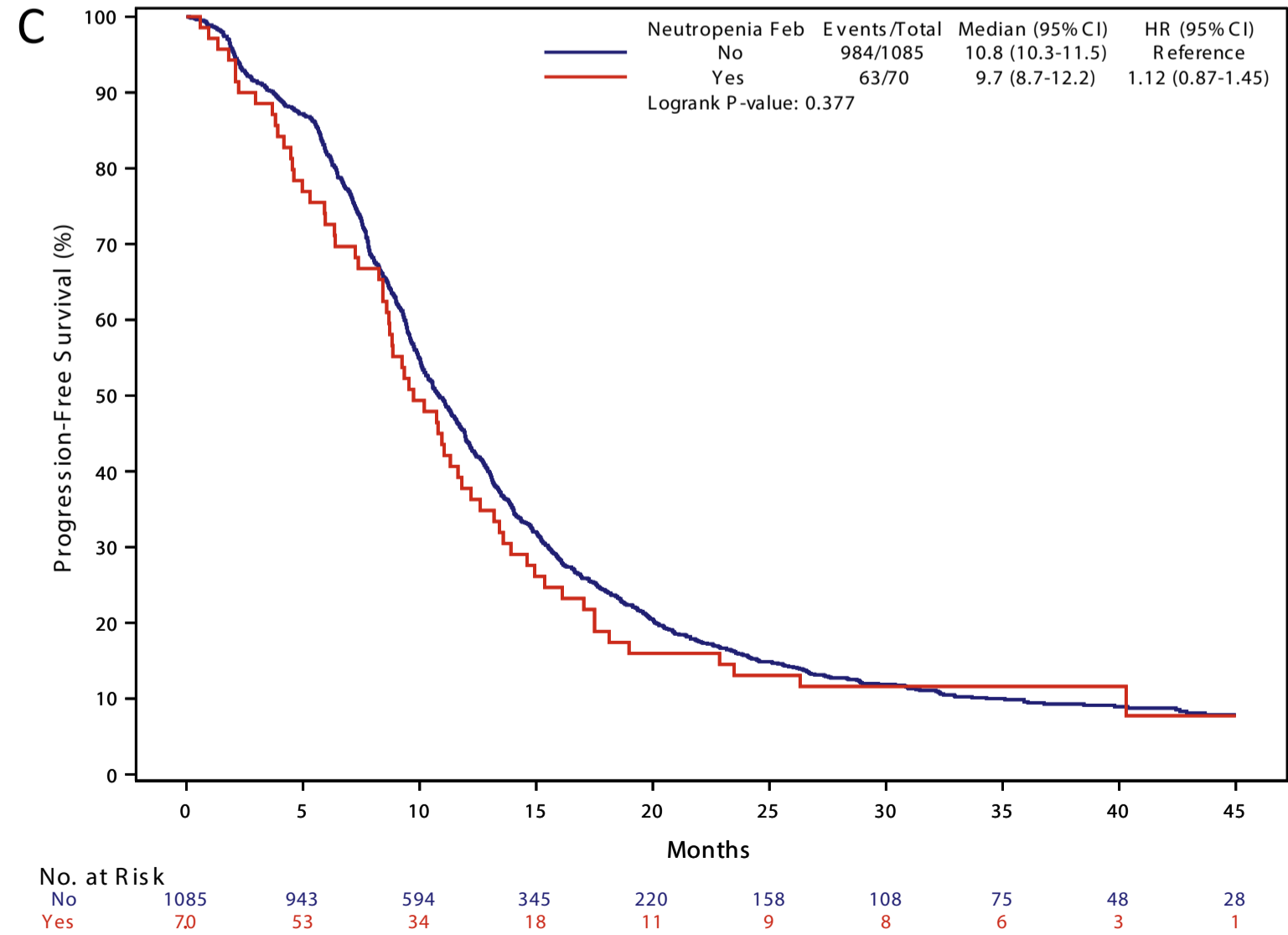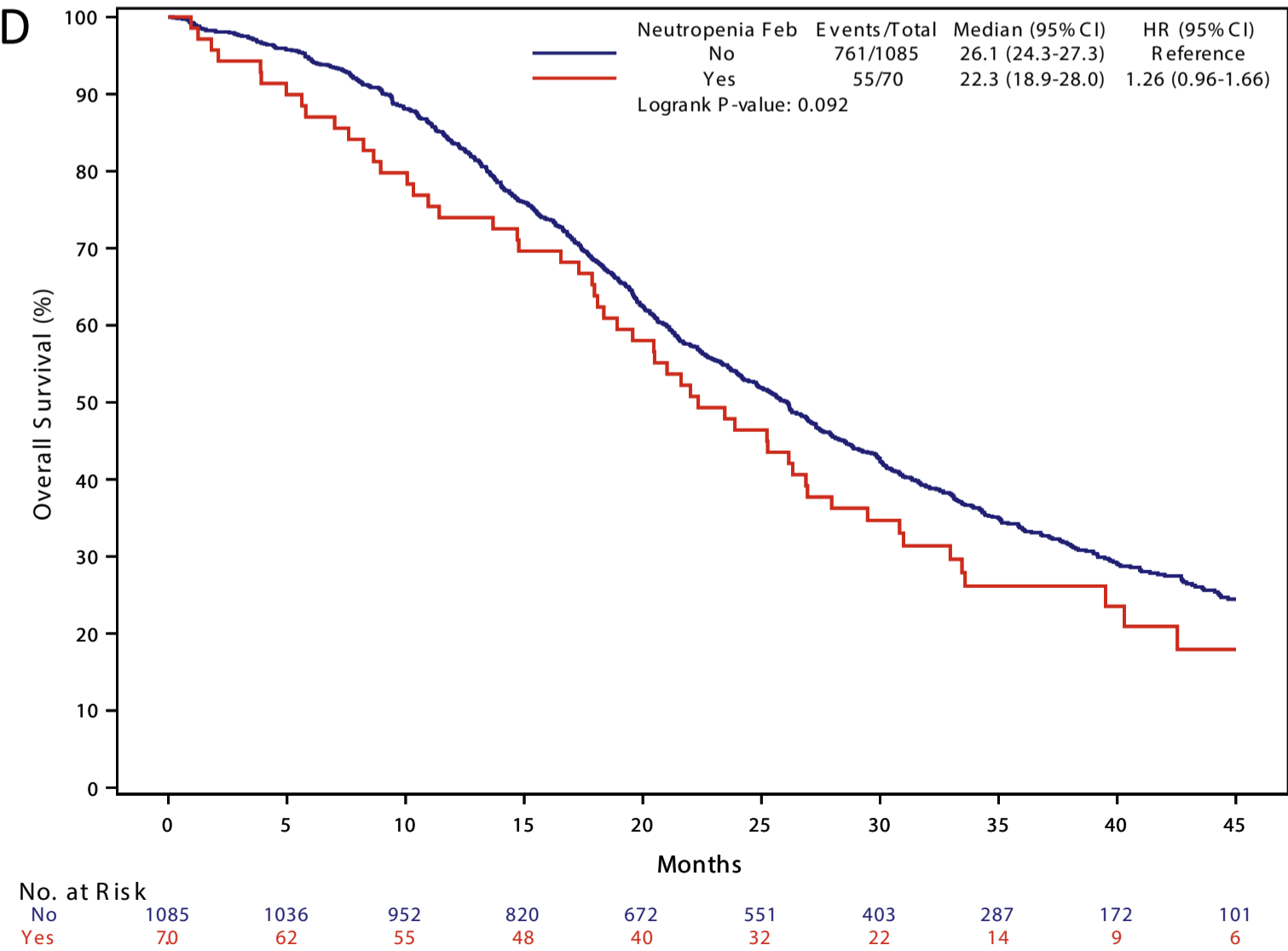

Supplement: Supplementary Figure S3 [file mmc4.pdf]
